# The Production of Clitic Pronouns: A Study on Bilingual and Monolingual Dyslexic Children

**DOI:** 10.3389/fpsyg.2018.02301

**Published:** 2018-11-27

**Authors:** Maria Vender, Shenai Hu, Federica Mantione, Denis Delfitto, Chiara Melloni

**Affiliations:** ^1^Department of Cultures and Civilizations, University of Verona, Verona, Italy; ^2^College of Foreign Languages and Cultures, Xiamen University, Xiamen, China; ^3^Department of Psychology and Cognitive Science, University of Trento, Trento, Italy

**Keywords:** clitic production, developmental dyslexia, bilingualism, morphosyntax, L2 exposure, bilingualism and dyslexia Interaction

## Abstract

Clitic production is reported to be challenging for impaired children, suffering from dyslexia or SLI, and for early second language learners too. On the contrary, research has not directly investigated the relation between dyslexia, bilingualism and clitic production. The aim of our study is that of addressing this topic, by analyzing the performance of 4 groups of children in a clitic elicitation task: 25 Italian monolingual dyslexic children (mean age 10;08 years old), 33 Italian monolingual typically developing children (9;99 years old), 25 bilingual dyslexic children with Italian as L2 (10;31 years old) and 31 bilingual typically developing children with Italian as L2 (10;30 years old). As inclusion criteria, bilingual children had at least 5 years of exposure to Italian, including 3 years of consecutive school attendance in Italy. Clitic production was assessed by means of an elicitation task in which the pronoun had to be produced either in the simple present or in the present perfect; higher difficulties were expected in this last condition, in which the clitic has to agree in gender and number with the past participle. Results revealed that dyslexic children, both monolingual and bilingual, performed worse than controls both in the simple present and in the present perfect, indicating that clitic production is challenging in dyslexia. As for the bilingual children, instead, differences were found between the two tasks. In the simple present, bilingual children performed very accurately and similarly to their monolingual peers, indicating that a target performance with clitics is accomplished by typically developing children with a longer exposure to Italian and suggesting that previously reported difficulties were related to linguistic immaturity and are likely to disappear as their L2 exposure and competence grow. In the present perfect, instead, both groups of bilinguals performed worse than their monolingual peers, suggesting that bilingualism could exacerbate the difficulties in the most challenging condition. Importantly, however, no negative effect of bilingualism in clitic production was found once controlling for the subjects' vocabulary, evidencing the importance of lexical competence in the target language for a native-like performance in clitic production.

## Introduction

It has been reported that Early Second Language (EL2) and bilingual children who are still acquiring their L2 can display difficulties in specific linguistic domains, including vocabulary and morphosyntax, and comprising the production of clitic pronouns (Paradis, 2005; Oller et al., 2007; Grüter and Crago, 2012). In the domain of language disorders, and specifically of Specific Language Impairment^1^ (SLI), several studies found that the acquisition of direct object clitic pronouns is strongly impaired and clitic production has been indicated as a good clinical marker for the identification of SLI in children (Bedore and Leonard, 2001; Bortolini et al., 2006; Stavrakaki et al., 2011; Arosio et al., 2014; Chondrogianni et al., 2015).

Clitic production is reported to be problematic also for children suffering from Developmental Dyslexia (DD) (Guasti, 2013; Zachou et al., 2013; Arosio et al., 2016; Mantione, 2016). However, the interaction between bilingualism and DD in clitic production has not yet been discussed. The aim of this study was to address this issue by analyzing the performance of monolingual and bilingual children, having or not a diagnosis of DD, in a clitic production task.

The paper is organized as follows. We first discuss the properties of clitic pronouns in Italian, focusing on the acquisition of clitics and on the studies reporting impaired clitic production in children with SLI, in children with DD and in EL2 individuals. We then briefly discuss the hypotheses elaborated to account for the difficulties in clitic production, maintaining that they may be related to processing factors. Finally, we present and discuss the results of our study.

### Direct-object clitic pronouns in italian: levels of complexity

Italian has three classes of pronouns, comprising strong pronouns, weak pronouns and clitics (Cardinaletti and Starke, 1999; Corver and Delfitto, 1999). Although Italian clitics include accusative, dative, genitive, partitive, locative and nominative clitics, we will focus only on accusative or direct object clitics (clitics henceforth) which have been investigated in this study.

Clitics present some levels of complexity which make them particularly difficult to acquire. At the phonological level, differently from other pronouns, clitics are unstressed monosyllabic morphemes and therefore they are said to be phonologically weak. Moreover, they are not phonologically independent, since they cannot occur in isolation, but they must be coupled with an adjacent verb. Depending on the position that they occupy with respect to the verb and on the finiteness of the verb itself, they can be proclitic (when they precede a finite verb), as in (1), or enclitic (when they follow a non-finite verb), as in (2).


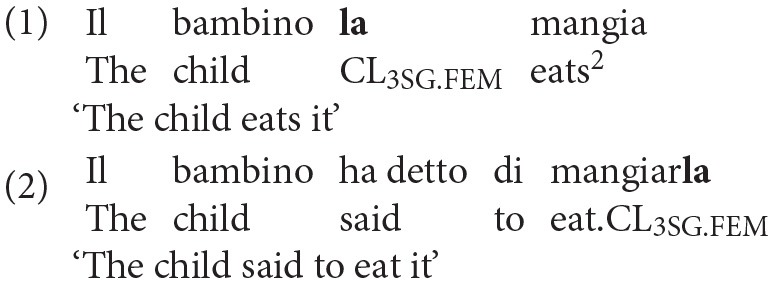


At the morphosyntactic level, clitic pronouns encode gender and number information in four different forms: *la* (feminine singular), *lo* (masculine singular), *le* (feminine plural) and *li* (masculine plural). In addition, both number and gender agreement are required with compound tenses, like the Italian *Passato Prossimo*, as reported in (3). Contractions of the singular clitics, both masculine and feminine, are commonly attested in Italian, as shown in (4); conversely, the contraction of plural clitics is ungrammatical, as displayed in (5).


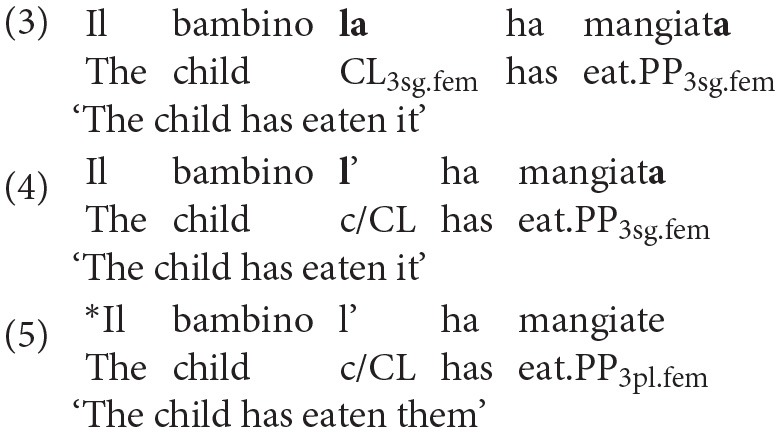


As already mentioned above, proclitics have a specific position in the sentence: they precede the predicate, moving from the canonical post-verbal position of internal arguments, and thus determining a non-canonical Subject Object Verb word order. At the syntactic level, clitics are the head of an impoverished DP (Determiner Phrase), originating as complements of the VP (Verb Phrase). Crucially, this head undergoes a complex movement operation, with further syntactic complications arising in the present perfect configuration (see Belletti, 1999). In this syntactic configuration, not only is the clitic pronoun moved to a (marked) preverbal position, but it also has to agree with the past participle, transmitting its gender and number features to the verbal form. As found by Moscati and Rizzi (2014), who compare the acquisition of different agreement configurations in Italian (Determiner-Noun, Subject-Predicate and Clitic-Past Participle), the clitic-past participle agreement configuration is the most complex one, as it implies the formation of a complex movement chain. Accordingly, it is mastered later by children acquiring L1 Italian, who still make errors at age four (Moscati and Rizzi, 2014)^3^. The authors argued that the reasons for the late mastery of this configurations might reside in the extra computational resources and processing costs imposed by these complex syntactic structures, requiring both a complex movement and an agreement operation (the point will be further elaborated in the section *Clitic Production, Working Memory, and Processing Abilities*).

Finally, from a pragmatic perspective, a clitic can be used appropriately only to refer to a salient antecedent, which must have been previously introduced in the discourse (Ariel, 1994). Importantly, only a sentence containing a clitic, like the one reported in (7), can be used felicitously to answer the question in (6), whereas the alternative sentence with the lexical or full DP, reported in (8), is infelicitous, though grammatically correct.


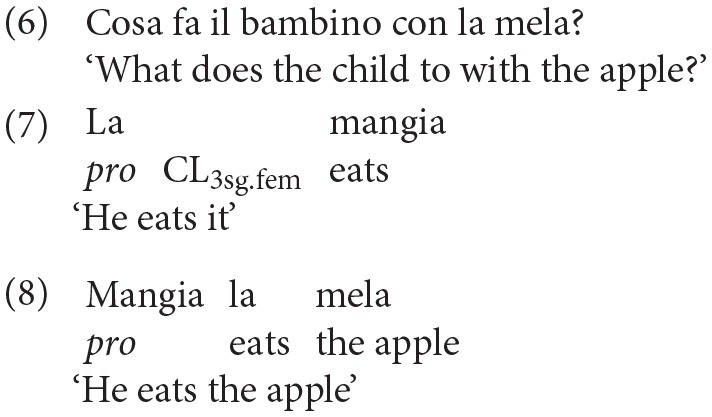


For the purposes of the present study, it can be useful to compare accusative clitics to reflexive clitics: similarly to accusative clitics, reflexive clitics occur in a preverbal position with finite verbs, as the result of a movement operation, but differently from accusative clitics, they are inflected neither for number nor for gender, resulting in the unique reflexive pronoun *si*, as in (9).


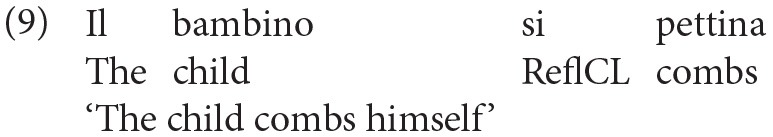


### The acquisition of clitic pronouns in typical and atypical development: SLI, dyslexic and unimpaired children

Normally developing and monolingual Italian children generally start to produce accusative clitics around 2 years of age, using them in an adult-like fashion, without displaying placement errors or replacing them with full pronouns (Guasti, 1993/1994; Caprin and Guasti, 2009; Moscati and Tedeschi, 2009). Moreover, past participle agreement under cliticization is correctly performed even from the youngest age, suggesting that agreement is successfully handled by young children (Belletti and Guasti, 2015).

Nevertheless, a stage of clitic omission has been reported in the literature, especially in spontaneous speech, suggesting that sometimes clitics are not produced in a context in which they would be expected. The omission stage is normally over at age 3–4, with constant progresses as they grow up (Leonini, 2006; Tedeschi, 2009). A stage of optional clitic omission has been reported also in other languages endowed with a clitic pronominal system, including European Portuguese (Costa and Lobo, 2007), Catalan (Wexler et al., 2004) and French (Pérez-Leroux et al., 2018). On the other hand, clitic omission was not reported in languages like Spanish (Wexler et al., 2004), Romanian (Babyonyshev and Marin, 2004), Greek (Tsakali and Wexler, 2003), and Serbo-Croatian (Ilic and Ud Deen, 2004).

This period of optional use of clitic pronouns is prolonged for children suffering from SLI, who display a marked tendency to omit clitics even at age 4–6, when their peers produce them in an adult-like fashion in the vast majority of the obligatory contexts (Bortolini et al., 2002, 2006). Interestingly, children with SLI produced much fewer clitics as compared with children who were 18 months younger than them (Leonard and Dispaldro, 2013). Furthermore, difficulties persist also in older children (mean age 7;3), who underperformed their chronological-age and grammatical-age matched peers (Arosio et al., 2014). However, their typical error is not clitic omission, as reported for preschool children, but the production of a full DP in (post-verbal) argument position instead of the clitic, which is grammatical, although not pragmatically appropriate.

Based on these studies, it has been proposed that the ability to produce accusative clitics can be used as a clinical marker for the identification of SLI in Italian, able to distinguish impaired children from age-matched normally developing children with high degrees of sensitivity and specificity (Bortolini et al., 2006; Arosio et al., 2014).

Difficulties with clitic production in SLI have been found crosslinguistically, with studies conducted in Greek (Chondrogianni et al., 2015), Romanian (Avram et al., 2013), Spanish (Bedore and Leonard, 2001), French (Hamann et al., 2003) and in other languages. Conversely, the production of reflexive clitics is reported not to be problematic for children with SLI, as found by Arosio et al. (2014), who reported low scores with object clitics but almost ceiling performance with reflexives, similarly to what found by Jakubowicz et al. (1998) for French.

Beyond these well-documented difficulties of clitic production in SLI, a number of recent studies has shifted the attention to Developmental Dyslexia (DD), using a very similar methodology and administering clitic elicitation tasks in which subjects were shown some pictures and told a short story and then asked to answer to a question eliciting the production of a sentence containing a clitic pronoun. DD is a disorder interfering with the acquisition of proper reading and spelling skills, in absence of other cognitive or physical disorders. Beyond the deficits in reading and writing, dyslexics typically exhibit linguistic deficits, especially in the phonological domain, but also in the comprehension and production of linguistically complex structures, as well as phonological short-term memory and working memory deficits (Ramus, 2003; Beneventi et al., 2010; Vender, 2017). Since clitic production is particularly complex from a morphosyntactic point of view, researchers have investigated how dyslexics performed in this task, reporting effective weaknesses. Specifically, 9-year old children with DD have been found to produce fewer clitics than age-matched controls, with a higher number of clitic substitutions, in which they produced a clitic wrongly inflected for gender or number, as *la* instead of *lo* (Guasti, 2013; Zachou et al., 2013). Besides assessing clitic production, Zachou et al. found that dyslexics were less skilled than controls in a grammaticality judgment task requiring them to detect omission errors.

Other studies focused on the processing resources involved in clitic production and investigated correlations between the subjects' linguistic performance and their working memory skills. To assess the impact of working memory (WM) in clitic production, Mantione (2016) developed a protocol aimed at testing clitic production across child populations, and at assessing the role of WM in this type of task. Specifically, Mantione examined clitic production in school-age dyslexics (mean age 9;4) compared to age-matched (mean age 9;4), grammar-matched (mean age 7;6) and younger controls (mean age 4;4). An elicited production task was used under two different levels of morphosyntactic difficulty and two different WM loads with the dual aim of (i) identifying and quantifying potential difficulties for dyslexics in the production of clitics and (ii) assessing whether sentence processing problems in dyslexia are more evident when WM demands are high. In this task, subjects were invited to produce sentences with a proclitic after seeing black and white drawings. Morphosyntactic difficulty was manipulated by eliciting sentences with clitic-past participle agreement [e.g., *L****i****ha inseguit****i***“(He) has chased them”] in addition to sentences without agreement [e.g., *Lo lava* “(He) is washing him”]. Moreover, WM load was manipulated by varying the delay between the presentation of the drawing (and the sentence that described it) and the question about that drawing. The overall results showed that dyslexics performed better than younger children, but significantly worse compared to both age-matched and grammar matched controls, uttering a wrongly inflected clitic, as in Zachou et al. (2013), or an indirect clitic instead of the target one. Further analysis demonstrated that the probability of producing a target clitic decreased as the WM load increased, and that this was statistically significant only for two of the four groups of participants: dyslexics and younger controls (Mantione et al., in preparation). The results suggest that the presence of dyslexics' difficulties in clitic production might be explained as resulting from their WM limitations.

Difficulties with clitic production in DD have been also reported by Arosio et al. (2016), who however found that dyslexics, instead of committing clitic substitution errors as reported by the studies reviewed above, tended to produce more full-DP structures in comparison to controls.

Moreover, Guasti (2013) and Arosio et al. (2016) noticed that some of the children with DD performed well in their clitic elicitation tasks, whereas some others (ranging from 25 to 40%) showed a particularly poor performance, scoring <1.5 SD below the mean score of control children. Based on these data, the authors hypothesized that the children showing a more severe difficulty with clitics and scoring lower than 1.5 SD below the mean actually suffered from an unrecognized form of SLI with an additional difficulty in reading, thus implying that the difficulties in clitic production found in dyslexia were actually related to the comorbidity of this disorder with SLI and not to dyslexia itself.

The relationship between SLI and DD in clitic production has been investigated by Avram et al. (2013), who compared monolingual Romanian children with dyslexia to children with SLI, to verify if it was possible to discriminate between the two disorders by looking at the typology of errors committed. They found that both groups of impaired children performed more poorly than controls, showing that clitic production is an area of weakness for both Romanian children with SLI and DD, although difficulties appeared to be more severe in SLI. However, the patterns of errors committed by the two groups were different: children with SLI produced a higher number of ungrammatical sentences (i.e., omitting the clitic or committing an agreement error), whereas dyslexics resorted more frequently to avoidance structures, uttering grammatical sentences with full DPs or with dative clitics instead of accusative clitics. The latter study suggests that clitic production is impaired also in dyslexic children without SLI and that despite the similarities between SLI and DD, it should be possible to distinguish between the two disorders by observing children's performance in clitic production and by considering in particular the typology of errors committed.

### The acquisition of clitics in early L2 and bilingual children

Clitics are especially difficult to acquire for EL2 individuals, both children and adults. A preliminary study conducted with preschool children acquiring Italian as their L2 and having Arabic as their L1 reported that EL2 children produced less target clitics in comparison to monolinguals, uttering a full DP in place of the pronoun (Guasti et al., 2013). This result echoes back to the study by Leonini and Belletti (2004), who tested adult L2 speakers of Italian with different mother languages confirming the presence of difficulties in clitic production and the tendency to omit the clitic or to produce a full DP instead of the target pronoun. A more recent study conducted by Vender et al. (2016) underlined the importance of taking into account the amount of exposure to the L2. Specifically, the authors assessed clitic production in the simple present in a group of 120 preschool children acquiring Italian as an L2 (mean exposure 3.5 years) and having Albanian, Arabic or Romanian as their L1, comparing their behavior to that of 40 age-matched monolingual Italian children. In order to analyse the performance of the subjects more in detail, precise information was gathered by means of a questionnaire collecting data about age of first exposure to Italian, quantity of exposure, traditional and cumulative length of exposure^4^. The competence in the L2 was assessed by means of a receptive vocabulary task (i.e., PPVT-R, Peabody Picture Vocabulary Test–Revised; Stella et al., 2000) and a comprehension task (i.e., a subset of the test *Comprendo*; Cecchetto et al., 2012). The authors found that EL2 children produced less target structures in comparison to monolinguals, suggesting that clitic production is difficult for children who are still acquiring Italian, as it is for children with language disorders. However, the most common error committed by the EL2 was not clitic omission, as is typical for preschool Italian monolingual children with SLI, but rather the production of a wrong clitic, with a prevalence of gender errors. Moreover, a correlation was found between the production of target clitics and the amount of exposure to Italian of the children, as well as with their competence in Italian, measured by the PPVT-R and the comprehension task. This suggests that EL2 with a higher exposure and a better competence in Italian were more accurate in clitic production, leading to the prediction that unimpaired bilingual children with a longer exposure should not exhibit difficulties in this domain, at least for what concerns the simple present, performing similarly to monolingual children. This prediction has been borne out by Vender et al. (2018), who found that 10-years-old typically developing children with on average 8 years of exposure to Italian performed very accurately and similarly to monolingual children in clitic production. Finally, all three groups of EL2 children manifested a similar behavior, independently from the L1 spoken, suggesting that their performance was not related to their L1.

The role of transfer from the L1 to the second language in clitic production has been tested also by Grüter and Crago (2012), who assessed clitic production and comprehension in EL2 children learning French as their L2 and having Spanish (a language with pronominal clitics disallowing the presence of referential null objects) or Chinese (a language without clitics but allowing the presence of null objects) as their L1. They found evidence for positive transfer in the rate of omissions, with an advantage in Spanish L1 over Chinese L1 children, but no evidence for negative transfer (omission errors were detected equally well by Spanish and Chinese children). Moreover, the authors found a correlation between WM and clitic production, indicating that performance in clitic production is linked to the subjects' WM and processing abilities, as will be discussed in the following section.

### Clitic production, working memory, and processing abilities

As discussed above, the production of clitics is particularly complex, especially for children with SLI, children with DD and EL2 children. One of the proposals developed to account for this difficulty attributes it to the phonological properties of clitics, which, being monosyllabic and unstressed, are poorly salient from the phonological point of view and hence difficult to acquire (Bortolini et al., 2006). However, this hypothesis is contradicted by the fact that reflexive clitics, which are as non-salient as object clitics, are acquired earlier and fully mastered by children with SLI and early L2 children.

A more accredited proposal argues instead that the difficulties in acquiring and mastering clitics are due to processing reasons. Prévost (2006) hypothesized that the production of accusative clitics is particularly costly in terms of processing resources, resulting, in the early stages, in a high rate of clitic omission. Specifically, he proposed that the computational complexity of clitics is related both to the projection of full-fledged representations and to the non-canonical position occupied by clitics as a result of syntactic movement [see Jakubowicz, 2011 for further refinements of the idea that movement implies (scales of) complexity relevant for understanding morphosyntactic development].

This hypothesis has gained further support by the study conducted by Grüter and Crago (2012), who tested EL2 children acquiring French as their L2 and gathered information about their processing abilities through a backward digit span task, which measures the subject's WM abilities. They found that WM was significantly correlated with omissions in clitic production, and that subjects with a lower WM capacity produced less target structures, omitting the pronoun more often. In their account, based on Ferreira's (2000) psycholinguistic model of incremental syntactic encoding, proclitic omissions might be due to a temporary overload of the WM resources needed to deal with the constituents in the syntactic workspace. This crowded workspace is due to the late transfer of the proclitic object to phonological encoding with respect to post-verbal objects, which can be instead immediately spelled out (see Grüter and Crago, 2012 for further details). Therefore, children with limited working memory resources might be unable to cope with the task of clitic production and, accordingly, omit the clitic pronoun.

A processing account for clitic production is in line with the results of acquisition studies, showing that young children can display problems in clitic production, which could be related to the fact that their processing abilities are still developing (Gathercole and Adams, 1993; Gathercole et al., 2004). Furthermore, this proposal can account for the difficulties in clitic production exhibited by children with SLI and DD: since both impairments are reported to be related to a WM inefficiency hampering their processing abilities (see Marinis, 2011 for SLI and Vender, 2017 for DD), it seems reasonable to suppose that the impaired children's poor performance with clitics is related to the complexity of the tasks, which exceeds their processing capacities, resulting in a lower number of target structures^5^.

Finally, this hypothesis also permits to explain why clitic production is difficult for children acquiring a second language: there is indeed evidence indicating that processing can be more expensive in a L2, in comparison to the L1 (Grüter and Crago, 2012)^6^. From this perspective, the morphosyntactic complexity of clitics can be more difficult to handle in a second language, requiring more processing resources especially in the first stages of its acquisition, and this can be taken to be responsible for the lower performances shown by EL2 and bilingual children with clitics.

## The current study

In the light of what discussed above, the present study aimed at assessing the production of clitics in DD and in bilingualism, to provide an answer to three research questions. First, we wanted to verify how children with DD performed in clitic production, while addressing also the role of WM in this task. Second, we aimed at verifying how bilingual children with a longer to Italian as their L2 performed in comparison to monolinguals in this task. Our last goal was to disentangle the relationship between bilingualism and dyslexia, assessing if bilingualism has an effect on dyslexia with respect to clitic production.

### Methods

#### Participants

One hundred and fourteen children with mean age of 10 years participated in our study, divided in four groups: 25 Italian monolingual dyslexic children (MD; mean age 10;08 years old), 33 Italian monolingual typically developing children (MC; 9;99 years old), 25 bilingual dyslexic children with Italian as L2 (BD; 10;31 years old) and 31 bilingual typically developing children with Italian as L2 (BC; 10;30 years old). One-way ANOVAs revealed no differences between the four groups regarding age, *F*_(3, 108)_ = .27, *p* = 0.84. A subset of the participants (all the monolingual and bilingual control children) were the same who took part in Vender et al.'s (2018) study. Children with DD were recruited from clinical speech centers or public schools in the area of Trento and Verona (Italy); they were diagnosed as having DD on standard criteria (ICD-10; World Health Organization, 2004) and they did not have diagnosed or reported oral language problems or hearing disorder. By controlling these aspects, we aimed at making sure, as far as possible, that our children did not suffer from SLI. Control children were recruited in the same schools as the dyslexic children, and they had no diagnosed or referred cognitive deficit, nor language problems, hearing disorders or reading difficulties.

For what concerns bilingual children, all participants acquired Italian as their second language and used a different language at home. We have decided not to restrict the choice of the L1 spoken by the subjects, due to the complexity of recruiting bilinguals with a diagnosis of DD and speaking the same L1^7^. However, we gathered complete information regarding the amount of exposure to both languages by using the Bilingual Language Exposure Questionnaire in Italian. The questionnaire was adapted from the Utrecht Bilingual Language Exposure Calculator (UBiLEC) (Unsworth et al., 2012) and was the same used in Vender et al. (2016). We collected information about the children's Age of First Exposure (AFE) to Italian, their current Quantity of Exposure (QE)^8^ to the L2, the Traditional Length of Exposure (TLE), which is calculated as the child's chronological age minus their age at first exposure to Italian, and the Cumulative Length of Exposure (CLE), which is a more precise measure considering other variables to determine the actual exposure to the L2. The data from one bilingual control child were missing, so only 55 bilingual children were analyzed. Their exposure to Italian is summarized in Table 1. One-way ANOVAs revealed no differences between BD and their BC regarding AFE, *F*_(1, 52)_ = 0.10, *p* = 0.75, QE, *F*_(1, 52)_ = 0.58, *p* = 0.45, TLE, *F*_(1, 52)_ = 0.16, *p* = 0.68, and CLE, *F*_(1, 52)_ = 0.14, *p* = 0.71.

**Table 1:** Means (SDs) of bilingual children's exposure to Italian.

	**AFE** **(in years)**	**QE** **(in percentage)**	**TLE** **(in years)**	**CLE** **(in years)**
Controls	2.24 (1.82)	0.64 (0.13)	8.08 (2.11)	2.39 (0.75)
Dyslexics	2.42 (2.31)	0.67 (0.14)	7.84 (2.25)	2.31 (0.81)

*AFE, Age of First Exposure; QE, current Quantity of Exposure; TLE, Traditional Length of Exposure; CLE, Cumulative Length of Exposure*.

All the children had normal or corrected to normal vision. The study was approved by the local ethics committee of the Department of Neurosciences and Movement Sciences of the University of Verona and it was conducted in accordance with the standards specified in the 2013 Declaration of Helsinki; moreover, we obtained written informed consent from the parents of all the children who took part in our research study.

#### Materials and procedures

All subjects were administered some preliminary tests in addition to the clitic production task, in order to collect specific measures concerning their cognitive level, reading abilities, receptive vocabulary and working memory. Each child was individually tested in a quiet room by the first author; the test session lasted approximately 45 min. All tests were coded twice by the first and the last author; the few disagreements were resolved after a discussion between the coders. A description of the tests is given below.

##### Non-verbal cognitive level

To assure that all participants had a normal cognitive level, we administered the *Raven's Colored Progressive Matrices* test (Raven et al., 1998); results were calculated as standard scores based on the Italian standardization (Belacchi et al., 2008). Children had to score within the normal ranges for their age in order to take part to the research.

##### Reading tasks

To participate in the study, dyslexics had to score 2 SD below the mean for their age/class of education in reading speed or accuracy of word or pseudo-word reading, as assessed by the test *Prova di lettura di parole e di nonparole* included in the *Batteria per la Valutazione della Dislessia e della Disortografia Evolutiva* (DDE-2; Sartori et al., 2007). Conversely, typically developing children had to score within the normal ranges in all reading tasks. In addition, we tested their ability of reading text using the Text Reading Task in the *Prove di Lettura MT per la Scuola Elementare-2* (Cornoldi and Colpo, 1998).

##### Receptive vocabulary

To have a standardized measure for the subjects' lexical abilities, we administered the *PPVT-R* by Dunn and Dunn (2000), adopting the Italian standardization by Stella et al. (2000).

##### Test of clitic production

Production of clitic pronouns was examined by means of an elicitation task, similar to those administered by Arosio et al. (2016) and Vender et al. (2016). During the task, subjects were shown some pictures displayed on a computer screen and told a short story that always involved one character doing something to one or two other characters, which was digitally recorded by a feminine Italian native speaker and played through loudspeakers connected to the pc.

When the first picture appeared, the characters of the story were introduced to the subject, while as the second picture appeared, the child was told that one character wanted to perform an action addressed to the other/s. After being shown the third picture, which portrayed the character performing that action, the child was invited to answer a question concerning what the character did and eliciting the use of a clitic. Since bilingual children typically have a less rich vocabulary in comparison to monolinguals, we tried to avoid possible lexical retrieval difficulties by selecting only very frequent and regular Italian verbs, which were, in addition, explicitly introduced in the story by the experimenter. All utterances were inserted in a supportive context in order to assure pragmatic felicity.

Our task elicited clitic production in two tasks: in the first one, the sentences were in the simple present (Italian *Presente*) and in the second one they were in the present perfect (Italian *Passato Prossimo*). In line with the considerations exposed in the introduction, we expected lower accuracy in the second task, where the past participle has to agree in number and gender with the clitic. A sample trial of both parts are reported below, respectively in (10, 11).

(10) Experimenter: “In questa storia ci sono un nonno e una bambina. La bambina sta uscendo di casa e il nonno non sa dove va. Il nonno vuole seguire la bambina. Cosa fa il nonno alla bambina?”
  ‘In this story there are a grandfather and a girl. The girl is leaving home and the grandfather doesn't know where she is going. The grandfather wants to follow the girl. What does the grandfather do to the girl?’
  Target answer: La segue.
  ‘He follows her.’
(11) Experimenter: “In questa storia ci sono una mamma e un ragazzo. Ieri il ragazzo è arrivato a casa presto e la mamma è stata contenta. La mamma voleva abbracciare il ragazzo. Cos'ha fatto la mamma al ragazzo?
  ‘In this story there are a mommy and a boy. Yesterday the boy arrived home early and the mommy was happy. The mommy wanted to hug the boy. What did the mommy do to the boy?’
  Target answer: Lo ha abbracciato.
  ‘She hugged him.’

We elicited 32 sentences each containing one of the four Italian accusative third-person clitics: *la* (feminine singular), *lo* (masculine singular), *le* (feminine plural) and *li* (masculine plural), 16 in the present and 16 in the present perfect. All verbs used in the task were obligatorily transitive, regular and highly frequent: *lavare “*to wash,” *salutare* “to greet,” *abbracciare* “to hug,” *accarezzare* “to caress,” *asciugare* “to dry,” *aiutare* “to help,” *spiare* “to peek at,” *vestire* “dress up,” *seguire* “to follow,” *bagnare* “to drench,” *tirare* “to pull,” *pettinare* “to comb,” *sgridare* “to scold,” *catturare* “to catch,” *spaventare* “to frighten” and *chiamare* “to call.” As underlined above, all these verbs have a regular past participle form in Italian.

In order to make the protocol as simple as possible, the characters involved in the stories were well known and highly stereotyped figures recurring throughout the task: four agents performing the different actions (a mother, a father, a grandmother and a grandfather) and eight patients undergoing the actions (a little boy, a little girl, a boy, a girl, two little boys, two little girls, two boys and two girls). Moreover, female agents were always paired with male patients and *vice versa*, in order to avoid confusion in the present perfect, where the verb has to agree with the clitic.

The 32 experimental trials were randomly ordered. The task was preceded by a familiarization section consisting of six training items; in the first and in the second training item, the child was told that a puppet would answer the questions and that she had to pay attention and to do the same with the remaining items. In the following four training items, the child was invited to answer the question; if she didn't produce a clitic pronoun, she was invited to do so by the experimenter. Conversely, no feedback was given in the experimental items.

The items were intertwined with 8 fillers (half in the simple present and half in the present perfect) eliciting the production of the reflexive clitic *si* “oneself” and involving the same characters and actions as the experimental items. The verbs used were taken from the list of the 16 verbs used throughout the experiment which allow a reflexive construction in Italian: *asciugarsi* “to dry oneself,” *lavarsi* “to wash oneself,” *vestirsi* “to dress oneself” and *pettinarsi* “to comb oneself.” An example of the simple present is provided below:

(12) Qui ci sono un bambino e una mamma. Il bambino si è sporcato e la mamma è arrabbiata. Il bambino vuole lavarsi. Cosa fa il bambino?
  ‘In this story there are a little boy and a mother. The little boy is dirty and the mother is upset. The little boy wants to wash himself. What does the little boy do?’
  Target answer: Si lava.
  ‘He washes himself.’

As already emphasized above, reflexive clitics are generally acquired earlier in comparison to accusative clitics, thus we didn't expect children to manifest difficulties with them.

Responses to the clitic elicitation task were coded in six categories for the Simple Present and nine categories for the Present Perfect, as schematically represented respectively in Tables 2 and 3.

**Table 2:** Response coding for the clitic task in the Simple Present.

**Category**	**Description**	**Example**
**Elicitation formula:** ***Cosa fa il nonno alla bambina?*** **('What does the grandfather do to the girl?)**		
*Target*	Sentence with the correctly inflected clitic	*La segue* ‘pro CL_3SG.FEM_ follows’
*Gender/number error*	Sentence with a clitic wrongly inflected for gender, number or both	*Lo segue* ‘pro CL_3SG.M_ follows’
*Omission*	Ungrammatical sentence with omission of the clitic	*Segue ‘*pro follows’
*Full DP*	Infelicitous sentence with a full DP in place of the clitic	*Segue la bambina* ‘*pro* follows the girl’
*Indirect clitic*	Sentence containing a dative clitic instead of the accusative one	*Le va dietro* ‘*pro* IndCL_3SG.FEM_ goes after’
*Other*	Irrelevant sentence	È* preoccupato* ‘pro is worried’

**Table 3:** Response coding for the clitic task in the Present Perfect.

**Category**	**Description**	**Example**
**Elicitation formula:** ***Cosa ha fatto il nonno alla bambina?*** **('What did the grandfather do to the girl?)**		
*Target*	Sentence with the correctly inflected clitic and the correct agreement with the past participle	*La ha seguita / L'ha seguita* ‘*pro* CL_3SG.FEM_ has followed’
*Gender/number error*	Sentence with a clitic wrongly inflected for gender, number or both	*Lo ha seguito* ‘*pro* CL_3SG.M_ has followed’
*Omission*	Ungrammatical sentence with omission of the clitic	*Ha seguito ‘*pro has followed’
*Full DP*	Infelicitous sentence with a full DP in place of the clitic	*Ha seguito la bambina* ‘*pro* has followed the girl’
*Indirect clitic*	Sentence containing a dative clitic instead of the accusative one	*Le è andato dietro* ‘*pro* IndCL_3SG.FEM_ has gone after’
*Other*	Irrelevant sentence	*Era preoccupato* ‘*pro* was worried’
*Non-target PP*	Sentence with contracted clitic and non-target past participle^a^	L'ha seguito ‘*pro* c/CL has followed_PL.FEM_’
*Agreement error*	Sentence with the correct clitic and a wrongly inflected past participle	*L**a** ha seguit**o** ‘*pro* CL_3SG.FEM_ has followed_SG.M_’
*Wrong contraction*	Sentence containing a contraction of the plural clitic	*L'ha seguite ‘*pro* c/CL has followed_PL.FEM_’

a *We included this category since in this case it is not possible to determine whether the clitic was wrong and agreed with the PP, or whether the clitic was correct and the PP was wrongly inflected*.

##### Working memory

As discussed above, working memory abilities are typically compromised in dyslexia. To verify the relationship between working memory and clitic production, following Grüter and Crago (2012) we administered a Backward Digit Span (BDS) task, measuring the children's WM (Baddeley, 1986, 2000). As in Grüter and Crago (2012) the task has been taken from the Working Memory Test Battery for Children (Pickering and Gathercole, 2001) and adapted to Italian (Vender, 2017). In the BDS, the experimenter uttered a sequence of digits of increasing length (from a minimum of 2 to a maximum of 7) and the child had to recall the digits in the reverse order, starting from the last digit heard and ending with the first. This test involves the simultaneous execution of two tasks: the subject has to store and recall the sequence of digits in forward order, as the experimenter presented it, and then she has to manipulate it in order to reproduce it in backward order. As a consequence, this task provides a measure of the children's working memory. All digits were uttered in even monotone at the rate of 1 per second. Each block was composed by 6 items; when the first four trials of one block were correctly recalled, the fifth and the sixth trials were omitted, and the child was presented with trials of the subsequent block. Testing stopped when the child committed three errors within the same block. The subject's span corresponded to the last block correctly recalled before stopping.

### Results

In this section we report the analyses of preliminary tasks and of the clitic production data, followed by the correlation analyses with language exposure and working memory. The data from one MD were discarded because of the lack of the data of WM, and the data from one BD child were excluded because of the low Raven score (<80). Thus, in total 112 children were included in the study, as shown in Table 4 for the group data of the children.

**Table 4:** Number, mean (SD) age in years, means (SDs) of z scores on the Raven and the reading tasks, and mean (SDs) of raw scores of the PPVT-R.

	**Monolingual dyslexics**	**Monolingual controls**	**Bilingual dyslexics**	**Bilingual controls**
No.	24	33	24	31
Age	10.02 (1.25)	9.99 (0.96)	10.24(1.29)	10.16 (1.24)
Raven	0.01 (0.75)	0.47 (0.79)	0.02 (0.66)	0.19 (0.82)
PPVT-R	99.96 (33.14)	102.69 (20.60)	86.45(22.56)	95.93 (13.30)
Word speed	−3.75 (2.73)	0.30 (0.64)	−1.81 (1.73)	0.25 (0.80)
Word accuracy	−2.20 (1.80)	0.31 (0.85)	−2.80 (1.32)	0.03 (0.94)
Non-words speed	−2.86 (2.58)	0.31 (0.62)	−0.64 (1.05)	0.63 (0.68)
Non-words accuracy	−2.13 (1.42)	0.32 (0.78)	−2.37 (1.10)	0.17 (0.82)
Text speed	−1.85 (1.80)	0.25 (0.39)	−1.01 (1.12)	0.14 (0.46)
Text accuracy	−1.12 (1.11)	0.48 (0.70)	−2.13 (1.12)	0.27 (0.50)

#### Preliminary measures

The results of the preliminary tasks are reported in Table 4. A series of one-way ANOVAs with Group as fixed factor and each measure as dependent variable were run. *Post hoc* Tukey tests were run when Group resulted significant.

##### Non-verbal intelligence

Group was not significant in the CPM Raven task [*F*_(3, 108)_ = 2.28, *p* = 0.08].

##### Vocabulary

One-way ANOVAs revealed no significant differences between the four groups regarding the scores on the PPVT-R, *F*_(3, 108)_ = 2.56, *p* = 0.06.

##### Reading tasks

First, regarding word reading, we found a main effect of Group in reading speed, [*F*_(3, 108)_ = 39.63, *p* < 0.001]; MD and BD were slower than MC and BC (*p* < 0.001), and MD were even slower than BD (*p* < 0.001). As for accuracy, Group was significant [*F*_(3, 108)_ = 44.27, *p* < 0.001], with both MD and BD reading significantly less accurately than the BC and MC (*p* < 0.001); moreover, BD were less accurate than MD (*p* < 0.001). Second, regarding non-words reading, one-way ANOVA revealed significant differences in their reading speed, *F*_(3, 108)_ = 34.26, *p* < 0.001, with the MD and BD reading significantly more slowly than the MC and BC (*p* < 0.001), and MD being even slower than BD (*p* < 0.001). Concerning accuracy, Group was significant [*F*_(3, 108)_ = 54.46, *p* < 0.001], with BD and MD reading significantly less accurately than the MC and BC (*p* < 0.001). Third, regarding text reading, we found significant differences in speed [*F*_(3, 108)_ = 25.42, *p* < 0.001], with MD and BD reading significantly more slowly than the MC and BC (*p* < 0.001), and MD reading even more slowly than BD (*p* < 0.05). We found a main effect of Group also in accuracy [*F*_(3, 108)_ = 55.73, *p* < 0.001], with MD and BD reading significantly less accurately than MC and BC (*p* < 0.001), and the BD reading significantly less accurately than all the other groups (*p* < 0.001).

### Clitic production

The experiment yielded 3,584 responses from children, including 3,141 target responses (88%). Monolingual dyslexic children performed better than bilingual dyslexic children (85 vs. 74%), while monolingual and bilingual controls performed nearly at ceiling (both about 93%). With respect to fillers (i.e., sentences with reflexive clitics), all the children responded 100% correctly.

Table 5 reports numbers, means and standard deviations (SDs) of different responses to the simple present and the present perfect in each group. As evident from the table, all children produced more target clitics in the simple present than in the present perfect. Dyslexic children made more errors than their control groups: in particular, they produced a higher number of *Gender/Number Errors* in the present (4 and 9% respectively; only around 1% for controls), with a prevalence for gender errors: monolingual dyslexics made 3.1% gender errors (e.g., *lo segue* instead of *la segue*), 0.3% number errors (e.g., *le segue* instead of *la segue*) and 1% gender + number errors (e.g., *li segue* instead of *la* segue), while bilingual dyslexics made 5.5% gender errors, 1.3% number errors, and 2.3% gender + number errors. There was no clear preference for masculine over feminine or for singular over plural.

**Table 5:** Mean (SDs) and Number (N/total score) of responses in the clitic production task for each group.

	**Monolingual dyslexics**	**Monolingual controls**	**Bilingual dyslexics**	**Bilingual controls**
**SIMPLE PRESENT**				
Target	0.88 (0.14) 336/384	0.95 (0.14) 503/528	0.79 (0.25) 299/384	0.94 (0.13) 465/496
Gender/Number error	0.04 (0.06) 17/384	0.01 (0.02) 2/528	0.09 (0.12) 35/384	0.01 (0.03) 5/496
Omission	0.02 (0.05) 7/384	0.00 (0.01) 1/528	0.03 (0.08) 13/384	0.01 (0.03) 6/496
Full DP	0.05 (0.10) 20/384	0.04 (0.14) 21/528	0.07 (0.14) 28/384	0.02 (0.05) 10/496
Indirect clitic	0.00 (0.01) 1/384	0.00 (0.01) 1/528	0.01 (0.03) 5/384	0.00 (0.00) 0/496
Other	0.01 (0.02) 3/384	0.00 (0.00) 0/528	0.01 (0.04) 4/384	0.02 (0.09) 10/496
**PRESENT PERFECT**				
Target	0.83 (0.17) 320/384	0.94 (0.13) 496/528	0.69 (0.23) 267/384	0.92 (0.13) 455/496
Gender/Number error	0.01(0.03) 5/384	0.00 (0.00) 0/528	0.05 (0.10) 20/384	0.00 (0.02) 2/496
Omission	0.03 (0.08) 10/384	0.01 (0.03) 3/528	0.05 (0.13) 18/384	0.01 (0.09) 8/496
Full DP	0.03 (0.06) 10/384	0.04 (0.09) 19/528	0.07 (0.11) 28/384	0.02 (0.03) 10/496
Indirect clitic	0.00 (0.00) 0/384	0.00 (0.01) 1/528	0.01 (0.02) 2/384	0.00 (0.01) 1/496
Other	0.01 (0.02) 2/384	0.01 (0.03) 4/528	0.02 (0.08) 9/384	0.01 (0.03) 5/496
Non-target PP	0.04 (0.07) 7/384	0.00 (0.02) 2/528	0.05 (0.05) 18/384	0.01 (0.03) 4/496
Wrong contraction	0.04 (0.07) 17/384	0.00 (0.01) 1/528	0.04 (0.07) 15/384	0.01 (0.02) 3/496
Agreement error	0.01 (0.02) 3/384	0.00 (0.02) 2/528	0.02 (0.03) 7/384	0.02 (0.05) 8/496

#### Statistical analysis

To address our research questions, we proceeded by firstly analyzing the data of the simple present, and then those of the present perfect. We started with an overall analysis, introducing Bilingualism (monolinguals vs. bilinguals) and Dyslexia (controls vs. dyslexics) as potentially significant fixed effect, and subjects and items as random effects. The dependent variables were *Target response*s and different error types, including *Gender/Number Error, Omission, Full DP, Non-Target PP, Wrong Contraction*, and *Agreement Error*. We did not analyze the *Indirect Clitic* and *Other* responses, as they were rarely produced. We fit our data to a series of mixed logit models using the *lme4* package in the R environment (R Development Core Team, 2017). The reference categories were bilingual children for Bilingualism and typically developing controls for Dyslexia. Effects were evaluated one by one on the basis of likelihood ratio tests; both first-level effects and interactions between the fixed-effect factors were examined (Baayen, 2008; Barr et al., 2013).

Second, we performed individual analyses. Following Arosio et al. (2016), we investigated individual differences by comparing the individual scores of dyslexic children to the mean scores of control children. For monolingual dyslexics and controls, we used the mean accuracy of monolingual controls as the reference mean score, and for bilingual dyslexics and controls, we used the mean accuracy of bilingual controls as the reference mean score, in order to identify the number of children whose scores were 1.5 SD below the reference mean score.

Third, we excluded these children and ran an analysis with Bilingualism (monolinguals vs. bilinguals) and Dyslexia (controls vs. dyslexics) as fixed effects, and subjects and items as random effects. As introduced earlier, Arosio et al. (2016) observed that some of their dyslexics performed very poorly (1.5 SD below the mean accuracy of the controls) and propose that they were actually not pure dyslexics but unrecognized SLI children. Our idea was to test their hypothesis by removing these potential SLI children from our groups of dyslexics and to verify if the effect of dyslexia remains.

Finally, having observed the presence of marginally significant differences among the four groups in the PPVT-R, we ran a further analysis to control for the role of vocabulary in the production of clitic pronouns, adding it as a covariate into a factorial model with Target clitics as dependent variable, to verify whether significant effects remained also after controlling this variable.

#### The analysis of the simple present

Using the *Target* responses as dependent variable, we found the main effect of Dyslexia (Wald *Z* = −3.78, *p* < 0.001), but neither the main effect of Bilingualism (Wald *Z* = 1.05, *p* = 0.29) nor the interaction between Dyslexia and Bilingualism (Wald *Z* = 0.40, *p* = 0.69) was found. This result suggests that children with DD were less likely to produce target clitics than their typical developing peers, irrespective of bilingualism.

We examined children's errors, including *Gender/Number Error, Omission* and *Full DP*. The main effect of Dyslexia was found when using *Gender/Number Error* and *Full DP* responses as dependent variable (Wald *Z* = 5.01, *p* < 0.001; Wald *Z* = −2.02, *p* < 0.05, respectively), while no significance was observed using *Omission* as dependent variable. Neither the main effect of Bilingualism nor the interaction between Dyslexia and Bilingualism were found using the error types as dependent variable (all *p* > 0.05). The results indicate that dyslexics produced more wrong clitics and full DPs in comparison to controls.

Then, we performed the individual analyses. MC produced target clitics in the simple present with a mean accuracy of 0.95 (*SD* = 0.14). By using this as the reference mean score, 3% (1 out of 33) MC and 8.3% (2 out of 24) MD scored <1.5 SD below the mean. On the other hand, BC produced target clitics in the simple present with a mean accuracy of 0.94 (*SD* = 0.13). By using this as the reference mean score, 6.5% (2 out of 31) BC and 25% (6 out of 24) BD scored <1.5 SD below the mean. This result confirms that dyslexic children had more difficulties in producing clitics as compared with their typical developing peers.

Furthermore, we ran an analysis by removing the 11 children who scored <1.5 SD below the mean scores of the controls. Using the *Target* responses as dependent variable, the main effect of Dyslexia was still observed (Wald *Z* = −3.41 *p* < 0.001), whereas neither the main effect of Bilingualism (Wald *Z* = 0.82, *p* = 0.41) nor the interaction between Dyslexia and Bilingualism were found (Wald *Z* = −0.13, *p* = 0.89). Regarding the error types, the main effect of Dyslexia was found, using *Gender/Number Error* as dependent variable (Wald *Z* = 3.22, *p* < 0.01), with no main effect of Bilingualism and no interaction (all *p* > 0. 05), while, interestingly, no significance was found using *Full DP*, which was instead reported in the previous analysis, and *Omission* as dependent variable (both *p* > 0.17).

#### The analysis of the present perfect

Using the *target responses* as dependent variable, we found the main effect of Bilingualism (Wald *Z* = −2.63, *p* < 0.01) and the main effect of Dyslexia (Wald *Z* = 5.36, *p* < 0.001), but not the interaction between them (Wald *Z* = 0.67, *p* = 0.50). The results indicate that children with DD were less likely to produce target clitics than their typical developing peers, and that bilinguals were less likely to produce target clitics than their monolingual peers.

We examined children's errors, including *Gender/Number Error, Omission, Full DP, Non-Target PP, Wrong Contraction* and *Agreement Error* responses. The main effect of Dyslexia was observed, when using *Gender/Number Error* (Wald *Z* = −3.29, *p* < 0.001), *Wrong Contraction* (Wald *Z* = −4.02, *p* < 0.001), *Non-Target PP* (Wald *Z* = −4.39, *p* < 0.001), and *Full DP* (Wald Z = −2.14, *p* < 0.05) as dependent variables. No significant differences were observed using the other error type as dependent variable. Neither the main effect of Bilingualism nor the interaction between Dyslexia and Bilingualism was found using the error types as dependent variable (all *p* > 0.05).

To run the individual analysis, we used the mean accuracy of MC's production of target clitics in the present perfect as the reference mean score for the monolingual children (*M* = 0.94, *SD* = 0.13) and we found that 9.1% (3 out of 33) MC and 29% (7 out of 24) MD scored <1.5 SD below the mean. For bilingual children, we used the mean accuracy of BC's production of target clitics in the present perfect as the reference mean score (*M* = 0.92, *SD* = 0.13). 3.2% (1 out of 31) BC and 35.8% (11 out of 24) BD scored <1.5 SD below the mean. Again, the results confirm that dyslexic children had more difficulties in producing clitics in the present perfect compared with their typically developing peers.

To verify if the effect of dyslexia remains even after having removed these children, we ran a further analysis by removing the 22 children who scored <1.5 SD below the mean scores of the controls. Using the *target responses* as dependent variable, we found again the main effect of Bilingualism (Wald *Z* = −2.34, *p* < 0.05) and the main effect of Dyslexia (Wald *Z* = −5.53, *p* < 0.001), with no interaction between them (Wald *Z* = 1.75, *p* = 0.08). With respect to the error types, neither the main effect of Bilingualism nor the interaction between Dyslexia and Bilingualism was found (all *p* > 0.05), whereas the main effect of Dyslexia was observed when using *Gender/Number Error* (Wald *Z* = 2.52, *p* < 0.05), *Wrong Contraction* (Wald *Z* = 2.05, *p* < 0.05), *Non-Target PP* (Wald *Z* = 2.77, *p* < 0.01) and *Full DP* (Wald *Z* = 2.12, *p* < 0.05) as dependent variables, as in the former analysis.

#### The analysis of the simple present with PPVT-R as a covariate

Given the marginally significant differences found amongst the four groups in receptive vocabulary, we decided to re-run the same analysis including the PPVT-R as a covariate to verify if the effects of Bilingualism and Dyslexia remained also once controlled for the subjects' receptive vocabulary. Using the *Target* responses as dependent variable, we found the main effect of Dyslexia (Wald *Z* = −3.14, *p* < 0.001), but neither the main effect of Bilingualism (Wald *Z* = 0.20, *p* = 0.84) nor the interaction between Dyslexia and Bilingualism (Wald *Z* = 0.02, *p* = 0.98) were found. This result suggests that children with DD were less likely to produce target clitics than their typical developing peers, irrespective of bilingualism. Moreover, we observed the significant effect of PPVT-R (Wald *Z* = 2.73, *p* < 0.01), showing that children's production improved as their vocabulary scores increased.

The same analysis was run also with the subset of children scoring higher than 1.5 SD below the mean of their reference category: again, the main effect of Dyslexia was still observed (Wald Z = −3.25 *p* < 0.001), whereas neither the main effect of Bilingualism (Wald *Z* = 0.09, *p* = 0.41) nor the interaction between them were found (Wald *Z* = −0.37, *p* = 0.71). The significant effect of PPVT-R was observed, too (Wald *Z* = 2.28, *p* < 0.01), revealing that children with better lexical abilities are more accurate in clitic production.

#### The analysis of the present perfect using PPVT-R as covariate

Using the *target responses* as dependent variable, we found the main effect of Dyslexia (Wald *Z* = −4.13, *p* < 0.001), but not the main effect of Bilingualism (Wald *Z* = 0.60, *p* = 0.55) and the interaction between them (Wald *Z* = 0.30, *p* = 0.76). We also observed a significant effect of PPVT-R (Wald *Z* = 2.73, *p* < 0.01), showing that children's production improved as their vocabulary scores increased. This result is particularly interesting, since it suggests that, once controlled for the subjects' vocabulary, the negative effect of bilingualism is not observed. This holds also for the analysis without the children scoring <1.5 SD below the mean scores of the controls. Indeed, we found again the main effect of Dyslexia (Wald *Z* = −5.08, *p* < 0.001), but neither the main effect of Bilingualism (Wald *Z* = 1.66, *p* = 0.10) nor the interaction between them (Wald *Z* = 1.27, *p* = 0.20). The effect of PPVT-R in this case tended to be significant (Wald *Z* = 1.78, *p* = 0.07).

### Bilingual children's language exposure and clitic production

**Figure 1:**
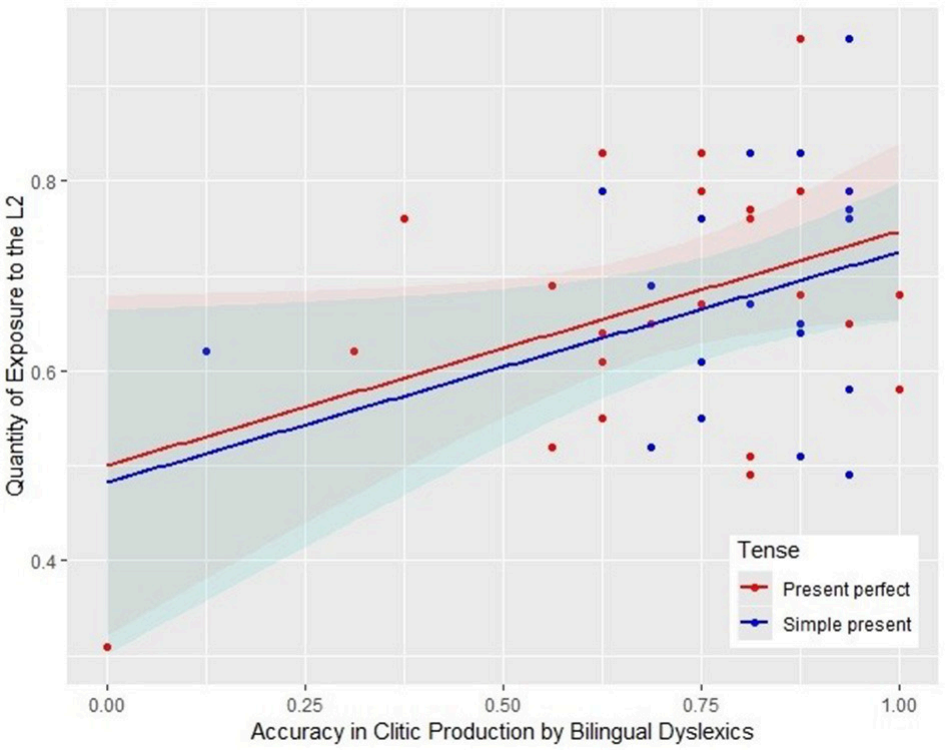
Scatter plot representing the relationship between target clitic production in both the present and the present perfect, and QE (Quantity of Exposure to the L2) in bilingual dyslexics.

We examined whether bilingual children's accuracy in clitic production was related to their exposure to Italian. As mentioned earlier, the data of one bilingual control child were missing, and thus in total 54 bilingual children were included in the correlational analysis. We found that for bilingual dyslexics QE positively correlated with target responses in the simple present (*r* = 0.43, *p* < 0.05) and in the present perfect (*r* = 0.44, *p* < 0.05), as shown in Figure 1. The results indicated that dyslexic children with a higher exposure to Italian produced more target responses in both tasks. No correlations were found instead among QE and clitic production for typically developing bilingual children.

### Working memory and clitic production

Finally, although a detailed explanation of the subjects' performance in terms of processing resources and limitations is not the main goal of our study, we wanted to verify whether the production of target clitics was related to the children's working memory abilities, as measured by their BDS.

The span of the four groups of children is shown in Table 6, where means and SDs are displayed; as it can be noticed, both groups of dyslexic children had a lower span in comparison to that of their typically developing peers. Using *BDS* score as dependent variable, the main effect of Dyslexia was found [*F*_(1, 108)_ = 10.86, *p* < 0.01], but neither a main effect of Bilingualism [*F*_(1, 108)_ = 0.05, *p* = 0.82] nor an interaction between Dyslexia and Bilingualism were found [*F*_(1, 108)_ = 0.00, *p* = 0.98]. These results confirm that dyslexic children's scores in the BDS were lower than those of their typically developing peers, in line with the studies mentioned above and reporting the existence of WM deficits in dyslexia.

**Table 6:** Means (and SDs) of the results in the backward digit span tasks for each group.

	**Monolingual dyslexics**	**Monolingual controls**	**Bilingual dyslexics**	**Bilingual controls**
BDS	2.67 (0.71)	3.21 (0.82)	2.71 (0.69)	3.26 (1.12)

**Figure 2:**
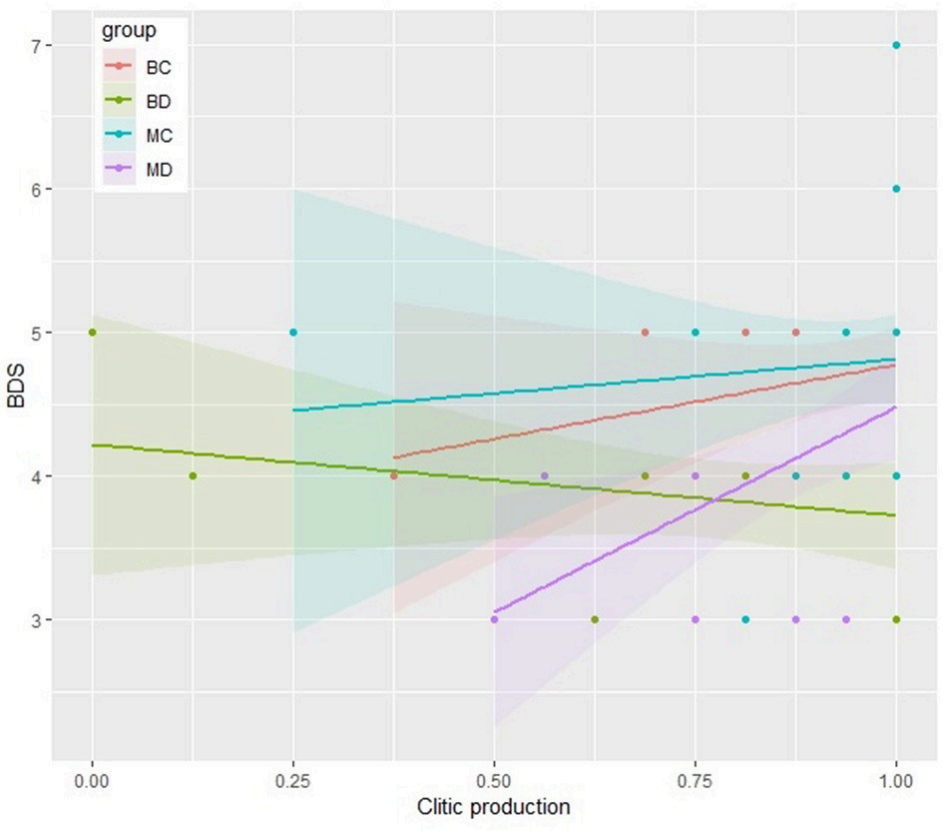
Scatter plot representing the relationship between production of target clitics in the Simple Present and BDS for each group.

**Figure 3:**
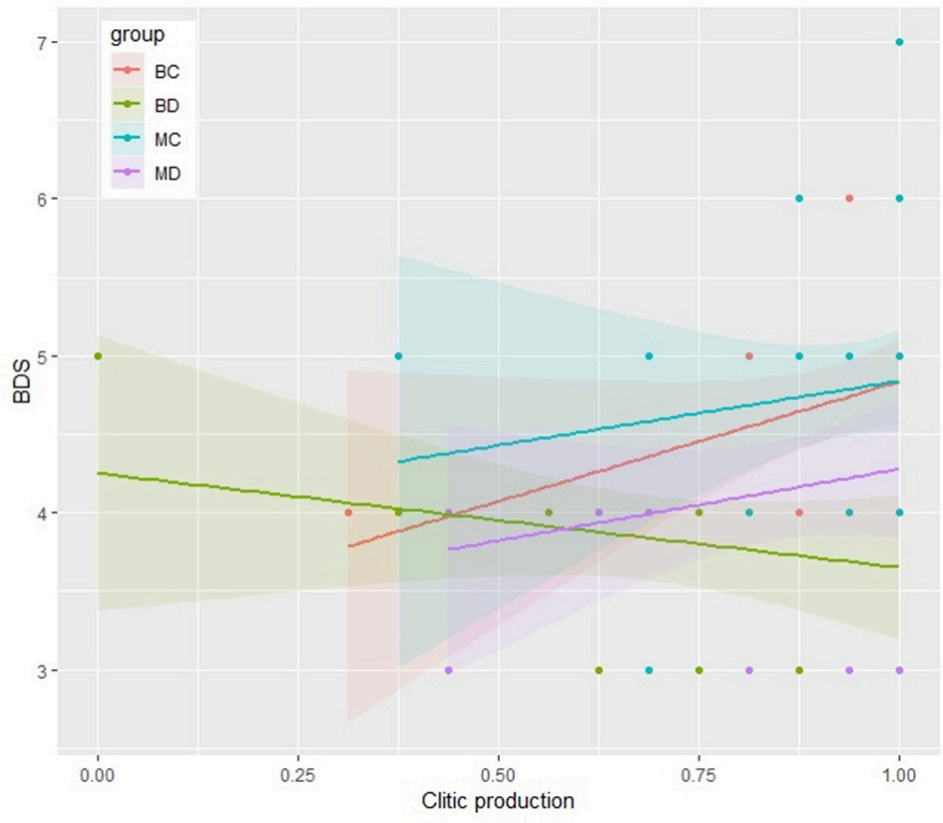
Scatter plot representing the relationship between production of target clitics in the Present Perfect and BDS for each group.

As displayed in the scatter plots reported in Figures 2 and 3, MD, MC and BC show a similar trend: accuracy in clitic production, indeed, increases as BDS scores increase, in both tasks. However, a significant correlation was found only for MD both in the simple present (*r* = 0.48, *p* < 0.05) and in the present perfect (*r* = 0.46, *p* < 0.05), and for BC, but limited to the Present Perfect (*r* = 0.37, *p* < 0.05). Conversely, BD show a different trend, displaying no correlations between BDS and accuracy in clitic production. Indeed, visual inspection of the scatter plots reveals that a few of the BD with a quite high BDS score had a very incorrect performance, thus suggesting that BD could rely on different processing strategies, not depending on WM, when engaged in the production of clitics.

## Discussion

This study aimed at comparing the performance of monolingual and bilingual children, with and without DD, in a task eliciting the production of clitic pronouns, which has been reported to be challenging for both dyslexics and EL2 children.

For convenience, we will discuss the results in distinct subsections.

### Clitic production and developmental dyslexia

As for our first research question, we found that children with DD, both monolinguals and bilinguals, performed worse in comparison to controls, both in the simple present and in the present perfect, confirming our predictions. Analysing the typology of errors committed by the children, we found that, as for the present, impaired children produced more incorrect clitics than controls, committing mainly gender errors. Moreover, dyslexics produced more full DPs than controls.

In the present perfect, instead, dyslexics produced more incorrect clitics than controls, more wrong contractions (e.g., ^*^L'ha seguite) and more non-target PP (e.g., *L'ha seguito* instead of *L'ha seguita*). Conversely, no differences were found among the four groups with respect to the other typologies of errors, including omissions and full DPs.

To address the issue raised by Guasti (2013) and Arosio et al. (2016), who suggested that the differences in clitic production exhibited by dyslexics could be due to the particularly severe deficits of a subset of children (ranging from 25 to 40% in their studies), possibly suffering from unrecognized SLI, we analyzed the individual performances of the children who took part in our study. As for monolingual dyslexics, we found that only a few subjects (ranging from 8.3 to 9.1%) scored <1.5 SD below the mean of the controls. The quite homogeneous performance of our dyslexics could be due to the fact that we carefully recruited only children who had not manifested oral deficits or received a diagnosis of SLI in preschool years, in order to exclude, as far as possible, a comorbidity with SLI. The proportion of children scoring lower than 1.5 SD below the mean of the control group was instead higher in bilingual dyslexics (ranging from 25 to 35.8%). This result seems to indicate that it can be more difficult to exclude a comorbidity with SLI in the recruitment of bilingual dyslexic children, even though it must be recognized that the higher variability found in this group is likely to be linked to individual differences related to exposure factors. This is also in line with the results of the correlation analysis, showing that bilingual dyslexics with a higher exposure to Italian had a more accurate performance.

Moreover, regarding the typology of errors, we found some interesting differences between the former analysis, including all subjects, and the latter, excluding the children with a possibly unrecognized SLI, especially with respect to the simple present. Specifically, in the second analysis we didn't find differences between dyslexics and controls in the production of full DPs in the simple present, which were instead detected in the former analysis. This is particularly interesting, since the (inappropriate) production of full DPs instead of clitics is the most typical error of school-aged SLI children. It seems thus reasonable to suppose that the children showing particularly severe deficits in clitic production were indeed cases of unrecognized SLI. Conversely, the production of full DPs remains significant in the present perfect also after removing the subjects scoring lower than 1.5 SD below the mean of their reference group, a result which is arguably related to the higher complexity of this condition.

Although we refrain from suggesting that clitic production should be used as a diagnostic test for the identification of dyslexia and we firmly believe that further studies are needed to investigate this issue, it is at least as important to emphasize that our findings show that the difficulties found in children with DD do persist even after removing these subjects from the analysis, thus suggesting that clitic production is actually compromised in dyslexia, and arguably not simply a side-effect of comorbidity with potentially unrecognized SLI.

Finally, it is worth noticing that the effect of dyslexia remains even after controlling for the subjects' vocabulary: this suggests that, despite the causal role played by lexical skills on the accuracy in clitic production, the difficulties exhibited by dyslexics are not entirely dependent on this measure.

### Clitic production and bilingualism

As for our second research question, we aimed at verifying how bilingual children performed in comparison to monolinguals in a clitic elicitation task. As discussed above, it is reported that bilingualism might have a negative effect in complex linguistic tasks, including the production of clitic pronouns. However, as Vender et al. (2016) pointed out, the difficulty found in the early stages of L2 acquisition seems to be related to the length of exposure to the L2, as well as to the children's competence in Italian. To test this prediction, we recruited a group of bilingual children with a longer exposure to Italian (8 years in average, including at least 3 consecutive years of school attendance in Italy). As expected, and extending the results by Vender et al. (2018) which were conducted only with unimpaired children, we found that BC showed an almost ceiling performance in clitic production in the simple present, approaching the monolingual standards and thus indicating that they had completely mastered it. Although performing worse than controls, bilingual dyslexics too performed similarly to their monolingual peers in this task.

This result confirms our predictions, suggesting that bilingual controls with a longer exposure to Italian do not display deficits in clitic production. Importantly, this holds for both impaired and unimpaired bilinguals, indicating that dyslexia does not interfere negatively with bilingualism on clitic production.

As for the present perfect, instead, although the accuracy of the typically developing bilinguals was still comparable to that of monolinguals, we found a negative effect of bilingualism for both groups of children. This result is arguably related to the higher complexity of sentences in the present perfect, in which the clitic has to agree in gender and number with the past participle, as discussed above. It may thus be the case that bilingual controls, despite having mastered clitic production in the simple present, still show problems in this task with respect to their monolingual peers.

Interestingly, however, it must be emphasized that the negative effect of bilingualism was not found, once vocabulary was controlled: this shows not only that having good vocabulary skills determines a better performance in clitic production, but also that the (lower) lexical abilities shown by bilingual children might be responsible for their (possible) difficulties in clitic production. This indicates, thus, that children with a higher competence in the L2 have a better performance in the task.

### Clitic production, bilingualism, and dyslexia

As for our third research question, we addressed the relationship between bilingualism and DD in clitic production. We found that bilingual dyslexics performed worse than monolingual dyslexics only in the present perfect, and that this difficulty disappeared once vocabulary was controlled for. Moreover, the two groups of dyslexic children committed very similar errors.^9^

All in all, our results suggest that the difficulties shown by bilingual dyslexics are related to dyslexia itself, and not to an alleged negative consequence of bilingualism in dyslexia. This is an important result confirming that bilingualism must not be seen as a factor hampering the acquisition of the second language in disordered children. It might happen, indeed, that parents of bilingual children with dyslexia are advised to abandon their mother tongue, in favor of a better development of the community language. However, our results indicate that this option should not be encouraged at all, since the difficulties experienced by bilingual dyslexics in some aspects of their linguistic competence, as in clitic production, would have not disappeared, had they been monolinguals.

### Clitic production and working memory skills

Finally, we aimed at assessing the correlation between accuracy in clitic production and working memory skills, as measured by the BDS task. No significant correlations were shown by monolingual controls, whereas a positive correlation was found for the bilingual controls, though limited to the present perfect. However, it must be noticed that both groups of controls showed a ceiling performance in the simple present, whereas bilinguals performed (slightly) worse in the present perfect. It thus seems that once a ceiling performance in clitic production is reached, as in both cases for monolingual controls, and in the simple present for bilingual controls, WM does not (or cannot) play a significant role. However, it still plays an important role when the competence is not optimal, as it is the case of the present perfect for BC, where they still display some difficulties. Consistently, a significant correlation was found in monolingual dyslexics, who showed a less accurate performance in both cases: in this case as well, children with higher WM skills were able to produce more target clitics.

Quite unexpectedly, instead, no correlations between WM and clitic production were found in bilingual dyslexics, indicating that WM skills are irrelevant for their performance, despite their poor accuracy in the task. This result seems to suggest that other factors might be responsible for these children's difficulties in clitic production, maybe including quantity of exposure to Italian, which was indeed significantly correlated to performance, or vocabulary (and more generally linguistic competence in the L2), which has been shown to be able to predict accuracy in clitic production.

In particular, we have noticed that some of the bilingual dyslexics showed a very inaccurate performance in both the simple present and the present perfect, despite displaying good BDS scores. This may indicate that they enact different processing strategies in comparison to the other groups of children when asked to produce clitics. This result echoes back the studies reported in the introduction (see footnote 6) in which it has been proposed that bilinguals may in some cases show a different processing in comparison to monolinguals (see Love et al., 2003; Garraffa et al., 2015, 2017). However, we believe that these results have to be complemented by further research in order to achieve a more complete comprehension of the role played by WM in clitic production.

Summarizing, the results of the correlation analysis offer only partial support of the hypothesis proposed by Prévost (2006), Grüter and Crago (2012), and Mantione (2016), according to which difficulties in clitic production are likely due to processing limitations, with subjects having better processing/WM capacities showing a more accurate performance. Even though an explanation in terms of processing resources does not seem to hold for bilingual dyslexics, it is in line with what we found for the monolingual dyslexics (and the bilingual controls in the task in which they display difficulties, i.e., in the present perfect). Nevertheless, as argued above, the absence of significant correlations in monolingual controls and in bilingual controls in the simple present could be due to the fact that the task is too easy for these subjects, who indeed display a ceiling performance. To verify this hypothesis, it would be interesting to assess the presence of correlations between WM and their performance in a more challenging task, involving a condition of working memory load, as in Mantione (2016).

Finally, observing the typology of errors committed by the children who took part in our study, we can speculate that the difficulty of clitic production lies in the selection of the appropriate gender (and number) features on the clitics; since this difficulty is not found with the past participle, as witnessed by the absence of differences in agreement errors, this might suggest that problems are related to the processing cost of the movement operation required by the clitic. More precisely, handling both movement and feature selection might exceed the capacities of children younger than 4 years old, children with SLI, children with dyslexia and EL2 individuals, giving rise, in our study, to a higher number of wrongly inflected clitics. Specifically, the children who took part in our research seemed to know that the clitic has to be produced in order to achieve pragmatic felicity (except for the infrequent cases of omission and production of full DPs, which moreover could be at least in part ascribed to the presence of children with SLI, as outlined above), and that it has to undergo a movement operation: they actually produced the pronoun in the correct position while in some cases failing to correctly specify the gender/number features of the clitic, either uttering an incorrect pronoun, or leaving it unspecified as in the case of wrong contractions and non-target past participle, which can be interpreted as an avoidance strategy.

## Conclusions, limitations and indications for future research

The goal of this study was to compare the performance of monolingual and bilingual children, with and without a diagnosis of DD, in a task eliciting the production of clitic pronouns. As expected, we found that both groups of dyslexics were less accurate in this task, underperforming in comparison to typically developing children. Conversely, no negative effect of bilingualism was found in the simple present, in which bilingual typically developing children showed a very accurate performance, reaching the monolingual standards. This indicates that bilingual children with a longer exposure to the L2 can achieve a complete mastery of complex structures such as clitic production in Italian, suggesting that the difficulties in clitic production typically reported in EL2 individuals are related to linguistic immaturity and are likely to disappear. As for the present perfect, bilingual controls show a poorer performance in comparison to their monolingual peers, suggesting that (slight) difficulties might remain in the most difficult condition; however, these difficulties disappear once vocabulary is controlled for, indicating that the bilinguals' poorer vocabulary might be responsible for their difficulties. The same holds also for bilingual dyslexics, who performed similarly to their monolingual peers, once controlling for their vocabulary.

A limitation of the present study lies in the fact that we tested children speaking different mother languages, some of which characterized by a clitic pronoun system similar to the Italian one (e.g., Spanish and Romanian), and some other lacking proclitic pronouns altogether (e.g., Arabic). Future research should address the issue of transfer from the L1 to the L2, by comparing monolingual children with two groups of bilinguals, whose L1 respectively has and doesn't have a pronominal clitic system (see Grüter and Crago, 2012). Finally, we found that an effect of dyslexia remains even after excluding the subjects showing a particularly poor performance and being potentially unrecognized SLI children. However, to address the issue concerning the relationship between DD and SLI more directly, we strongly encourage future research to focus on the comparison of three distinct groups of subjects, including children with pure DD, children with pure SLI and children displaying both disorders, to ascertain the presence of differences or similarities in clitic production among these populations.

## Author contributions

MV conceived the project with support of CM, FM, and DD. MV designed the experiment, collected the data and wrote the first draft of the paper. SH ran the statistical analysis and wrote the Results section of the paper. MV and CM interpreted the results with support of DD and FM. All the authors revised the work critically for important intellectual content and gave the final approval of the version to be published.

### Conflict of interest statement

The authors declare that the research was conducted in the absence of any commercial or financial relationships that could be construed as a potential conflict of interest. The reviewer ML and handling Editor declared their shared affiliation.

## Footnotes

^1^SLI is a neurodevelopmental disorder characterized by the presence of linguistic deficits, affecting in particular the lexical and morpho-syntactic domain, in children with a normal cognitive level and no physical or neurological disorders (Leonard, 2014). It must be emphasized that there is disagreement about the terminology to be used to refer to children suffering from language disorders: the term Primary Language Impairment (PLI), referring to children exhibiting an impaired linguistic competence together with other cognitive or physical deficits, is also used (see Reilly et al., 2014 for an extensive discussion on the current debate). In this paper, we employ the term SLI, to refer to previous studies reported in the literature and addressing clitic production in children with SLI.

^3^See also Pirvulescu and Belzil (2008) for a paper investigating clitics and past participle agreement in French and showing that three years old children do not mark past participle agreement.

^4^Traditional length of exposure (TLE) is calculated as the child's age at first exposure to the L2 subtracted to her chronological age, whereas the cumulative length of exposure (CLE) is a more complex measure that takes into account other variables to determine the real exposure to the second language over time (languages spoken to and by the child with parents and siblings, at school, in extra-curricular activities and during holidays; for more details, see Unsworth et al., 2012).

^5^A different explanation in terms of processing is offered by Moscati and Rizzi (2014), who propose a syntactic source for the computational complexity of the Clitic-Past Participle agreement configurations in Child Italian. Adopting a minimalist approach to syntactic derivations, they postulate that the reason for the computational load associated with clitic constructions might reside in the intervention of a *phase* edge, distinguishing Cl-Past Participle agreement from other configurations in which arguably no phase edge is crossed (Chomsky, 2001).

^6^Other accounts have also been proposed to explain the presence of differences in linguistic tasks between monolingual and bilingual children. The processing strategies adopted by bilinguals could indeed be simply different from those accessed by monolinguals, as theorized by Love et al. (2003), who argued that models of language processing only based on studies conducted on monolinguals could not be appropriate to account for the different performance of bilingual children. A similar observation was also put forward by Garraffa et al. (2015, 2017), who explained the better performance in the comprehension of complex relative clauses by Sardinian-Italian bilingual children with respect to monolinguals by arguing that bilinguals might be endowed with different grammatical representations for these sentences.

^7^The L1 spoken by the bilingual dyslexics were Arabic (7 children), Albanian (7), Romanian (5), Spanish (2), Hindi(1), Turkish (1), Yoruba (1) and Senegalese Wolof (1). The L1s spoken by the bilingual controls were Romanian (10 children), Arabic (8), Albanian (4), Hindi (2), Spanish (1), Ghanaian English (1), Yoruba (1), Moldovan (1), Serbian (1), Polish (1) and Macedonian (1). All of these languages, except for English, for which we had only one bilingual speaker, have a clitic pronominal system, thus suggesting that, despite the heterogeneity of the L1s spoken by the subjects, there is homogeneity among the two groups at this respect. Notice, moreover, that no interference effects from the L1 to the L2 should be expected according to the literature reviewed above.

^8^Quantity of Exposure has been calculated considering the languages at which the child is exposed at home, at school and during possible extra-curricular activities; both the languages spoken by the child and those the child is spoken to are taken into account (see Unsworth, unpublished, for a detailed description of the variables considered and the algorithms used). Notice that the information gathered by means of the questionnaire refers almost exclusively to the oral exposure of the children, whereas written exposure is indeed not explicitly considered. However, since our four groups of participants attend the same Italian schools, it can be argued that they are receiving the same amount of exposure to written language at school and in their homework.

^9^One could object that the worse performance shown by bilingual dyslexics might be due to different degrees of severity of the disorder. Although it might be reasonable to argue that children with a more severe form of dyslexia could perform worse in clitic production, we do not think that this can explain their lower accuracy in clitic production: our bilingual dyslexics, indeed, read less accurately existing words and text than their monolingual peers, which has been already reported in the literature and explained as a consequence of their lower lexical abilities (see Scortichini et al., 2012, for similar results in Italian), but they were actually faster than monolingual dyslexics in all reading measures.
